# Reference gene selection and myosin heavy chain (MyHC) isoform expression in muscle tissues of domestic yak (*Bos grunniens*)

**DOI:** 10.1371/journal.pone.0228493

**Published:** 2020-02-06

**Authors:** Xiaoyun Wu, Xuelan Zhou, Xuezhi Ding, Min Chu, Chunnian Liang, Jie Pei, Lin Xiong, Pengjia Bao, Xian Guo, Ping Yan

**Affiliations:** 1 Key Lab of Yak Breeding Engineering, Gansu Province, Lanzhou, China; 2 Lanzhou Institute of Husbandry and Pharmaceutical Sciences, Chinese Academy of Agricultural Sciences, Lanzhou, China; Universidade Federal de Viçosa, BRAZIL

## Abstract

Domestic yak (*Bos grunniens*) is the most crucial livestock in the Qinghai-Tibetan Plateau, providing meat and other necessities for local people. The skeletal muscle of adult livestock is composed of muscle fibers, and fiber composition in muscle has influence on meat qualities, such as tenderness, pH, and color. Real-time quantitative polymerase chain reaction (RT-qPCR) is a powerful tool to evaluate the gene expression of muscle fiber, but the normalization of the data depends on the stability of expressed reference genes. Unfortunately, there is no consensus for an ideal reference gene for data normalization in muscle tissues of yak. In this study, we aimed to assess the stability of 14 commonly used candidate reference genes by using five algorithms (GeNorm, NormFinder, BestKeeper, Delat Ct and Refinder). Our results suggested *UXT* and *PRL13A* were the most stable reference genes, while the most commonly used reference gene, *GAPDH*, was most variably expressed across different muscle tissues. We also found that the *extensor digitorum lateralis* (EDL), *trapezius pars thoracica* (TPT), and *psoas major* (PM) muscle had the higher content of type I muscle fibers and the lowest content of type IIB muscle fibers, while *gluteobiceps* (GB) muscle had the highest content of type IIB muscle fibers. Our study provides the suitable reference genes for accurate analysis of yak muscle fiber composition.

## Introduction

The yak (*Bos grunniens*) is one of the livestock that can adapt to the harsh environment of the Qinghai-Tibetan Plateau and adjacent Alpine regions, which provides meat, milk, hair, transport and fuel for residents [1]. There are around 14 million yaks in the world, 95% of them are distributed in China [2]. As the consumer demand for high quality meat products continues to increase, there is a growing demand for high quality yak meat in most areas of China. Therefore, understanding the characteristics of meat quality is a major priority in yak production.

Skeletal muscle is a heterogeneous tissue that consists of a large variety of physiologically and biochemically diverse fiber types [3]. According to the different structure, contractile properties, metabolic and morphological traits, skeletal muscle fibers of adult livestock are classified into four types: type I (slow-twitch, oxidative metabolism), type IIA (fast-twitch, oxidative metabolism), type IIB (fast twitch, glycolytic metabolism) and type IIX (fast-twitch, intermediate metabolism) [4]. Many studies indicate that muscle fiber type composition has profound influence on the biochemical properties of the muscle, and the meat quality traits of livestock [5,6]. Muscle fiber type composition is commonly used to predict the meat quality traits of livestock. Myosin heavy chain (MyHC) is the most abundant muscle structural protein, comprising about 35% of the protein pool [7]. Four adult MyHC isoforms (I, IIA, IIX and IIB) have been identified in the skeletal muscle of representative mammalian [8]. The slow-twitch muscle fibers of skeletal muscle primarily express the MyHC I isoform, while fast-twitch muscle fibers express other faster isoforms (MyHC 2A, MyHC 2X and MyHC 2B) [9]. Therefore, muscle fiber types are characterized by isoforms of the MyHC genes which are expressed. The composition of each muscle fiber type can generally be determined by the protein and mRNA expression of four MyHC isoforms using immunohistochemistry [10], SDS-PAGE [11], in situ hybridization [12], and Reverse transcription PCR (RT-PCR) approaches [13], but these approaches are semiquantitative. Currently, the real-time quantitative polymerase chain reaction (RT-qPCR) has become a powerful method used to analyze the muscle fiber composition due to its high sensitivity, specificity, simplicity, and reproducibility [14].

RT-qPCR is considered as the gold standard for defining accurate expression profiles of selected genes [15,16]. The reliability of RT-qPCR results can be easily influenced by several variables, including the initial sample amount, RNA integrity and quantity, accurate reverse transcription, and primer efficiency. To minimize the influence of these factors, reference genes are usually used to calibrate and normalize the expression level of target genes [17]. Ideal reference genes should have a ubiquitous expression and very low variance across various development stages, types of tissues, environmental conditions, and health conditions [18]. Recently, some commonly used reference genes for RT-qPCR have been analyzed in different tissues of yak [19–22]. Unfortunately, as of now, there are no available endogenous control genes for RT-qPCR data normalization that have been identified for various muscle of yak.

The aim of this study was to select and evaluate the stable reference genes that can be used to normalize mRNA expression by RT-qPCR in skeletal muscle tissue of yak. To attain this aim, we selected 14 commonly used candidate reference genes from published literature for gene expression studies. The expression stabilities of these reference genes were analyzed using the GeNorm [23], NormFinder [24], BestKeeper [25], Delat Ct [26] and Refinder programs [27]. Additionally, the validated reference genes were used to identify the muscle fiber composition in ten skeletal muscles of yak.

## Materials and methods

### Muscle samples

Three male Ashidan yaks were raised at the Qinghai Datong yak farm of Province with the same feeding conditions. Yaks were slaughtered at the body weight of 176.00 ± 8.54 kg (30 months of age). After slaughter, ten different skeletal muscles were immediately frozen in liquid nitrogen and stored at -80 °C until further processing. These ten skeletal muscles were *extensor digitorum lateralis* (EDL), *psoas major* (PM), *latissimus dorsi* (LD), *gluteobiceps* (GB), *gastrocnemius* (GC), *fibularis longus* (FL), *semitendinosus* (ST), *trapezius pars thoracica* (TPT), *supraspinatus* (SP) and *longissimus dorsi* muscle (LDM). This study was carried out in strict accordance with the recommendations in “Guidelines for Experimental Animals” of the Ministry of Science and Technology (Beijing, China), and all of the experimental protocols and procedures were approved by the Animal Administration and Ethics Committee of Lanzhou Institute of Husbandry and Pharmaceutical Sciences of CAAS (Permit No. SYXK-2014-0002).

### RNA extraction and cDNA synthesis

Total RNA was isolated using the animal tissue RNA isolation kit (ZDGSY, Beijing, China) following the manufacturer’s protocol. RNA concentration and purity (A_260_/A_280_) was determined using NanoDrop2000 spectrophotometer (Thermo Fisher Scientific, Waltham, MA, USA). Integrity of RNA was evaluated using 1% agarose gel. Total RNA (1000 ng) was reverse transcribed according to the instructions of PrimeScript^™^ RT reagent kit with gDNA Eraser (TaKaRa, Dalian, China). The cDNA was stored at -20 °C until required.

### Primers design

Based on literature review, the following genes were chosen to evaluate using the RT-qPCR method for expression of stability: glyceraldehyde-3-phosphate dehydrogenase (*GAPDH*), actin-beta (*ACTB*), ubiquitously expressed prefoldin-like chaperone (*UXT*), TATA box binding protein (*TBP*), tyrosine 3-monooxygenase/tryptophan 5-monooxygenase activation protein zeta (*YWHAZ*), ribosomal protein L13a (*RPL13A*), dehydrogenase complex subunit A (*SDHA*), ribosomal protein S15 (*RPS15*), hypoxanthine phosphoribosyl transferase 1 (*HPRT1*), peptidylprolyl isomerase A (*PPIA*), hydroxymethylbilane synthase (*HMBS*), mitochondrial ribosomal protein L39 (*MRPL39*), beta-2-microglobulin (*B2M*) and protein phosphatase 1 regulatory inhibitor subunit 11 (*PPP1R11*) (S1 Table). Among these candidate reference genes, *PPP1R11*, *UXT*, *MRPL39*, *RPS15*, *HMBS*, *YWHAZ*, *TBP* and *ACTB* have been reported to be optimal reference genes for normalization in yak [19–22]. In addition, *HPRT1*, *PPIA*, *SDHA*, *B2M* and *RPL13A* were identified as reference gene for expression studies in muscle tissue of pig, chicken and human [28–31]. *GAPDH* have been utilized as reference gene in numerous studies of yak. Primers for *TBP*, *ACTB*, *PPIA*, *HPRT1*, *GAPDH* and *SDHA* were used from Li et al [21]. The sequences of other genes were obtained from NCBI. All the primer pairs were designed by Primer 5.0 software with the length of 20 ± 3 bases, and size of amplicon ranging from 79–198 bp (Table 1). Primer specificity of each reference gene was confirmed by melting curve analysis and 1.5% agarose gel electrophoresis.

**Table 1:** The primers and amplification characteristics of 14 candidate reference genes and a target gene.

Gene Name	Accession No.	Primer Sequence (5’-3’)	Amplicon Size (bp)	E (%)	R^2^
**ACTB**	XM_005887322.2	F: ATTGCCGATGGTGATGAC	177	93.0	0.9921
		R: ACGGAGCGTGGCTACAG			
**GAPDH**	XM_014482068.1	F: TCACCAGGGCTGCTTTTA	126	97.0	0.9973
		R: CTGTGCCGTTGAACTTGC			
**UXT**	XM_005899362.2	F: AGGTGGATTTGGGCTGTAAC	170	104.0	0.9999
		R: CTTGGTGAGGTTGTCGCTGA			
**TBP**	XM_005908677.2	F: GTCCAATGATGCCTTACGG	82	95.0	0.9936
		R: TGCTGCTCCTCCAGAATAGA			
**YWHAZ**	XM_005887010.2	F: AATGTTGTAGGAGCCCGTAG	190	95.0	0.9999
		R: CTGCTTGTGAAGCGTTGG			
**RPL13A**	XM_014481217.1	F: CAAGCGGATGAACACCAA	192	92.0	0.9997
		R: GCAGCAGGAACCACCATT			
**SDHA**	XM_005894659.2	F: GGGAACATGGAGGAGGACA	188	104.0	1.0000
		R: CCAAAGGCACGCTGGTAGA			
**RPS15**	XM_005890466.2	F: GACCTTCCGCAAGTTCACCT	198	101.0	0.9999
		R: ACCACCTCGGGCTTCTCCAT			
**HPRT1**	XM_005911180.2	F: GTGATGAAGGAGATGGG	79	109.0	0.9999
		R: ACAGGTCGGCAAAGAAC			
**PPIA**	XM_005891872.2	F: TTTTGAAGCATACAGGTCC	98	94.0	0.9999
		R: CCACTCAGTCTTGGCAGT			
**HMBS**	XM_005897126.2	F: GAACAAAGGAGCCAAGAAC	121	103.0	0.9997
		R: CAGAGGGCTGGGATGTAG			
**MRPL39**	XM_005898618.2	F: AAACCTTTGACCAAGTCCTGT	135	95.0	0.9975
		R: TTCCTCTTTGAATGCCCTCTC			
**PPP1R11**	XM_005911410.2	F: CAGAAAAGACAGAAGGGTGC	164	99.0	0.9999
		R: TTCCGAAGTTTGATGGTTAG			
**B2M**	XM_005911364.2	F: CTGAGGAATGGGGAGAAG	80	95.0	0.9923
		R: TGGGACAGCAGGTAGAAA			

### RT-qPCR analysis

All RT-qPCR analyses were conducted in 96-well plates using the BioRad CFX96^™^ Real-Time PCR system (Bio-Rad Laboratories, CA, USA). The PCR program for amplification was as follows: an initial denaturation step of 3 min at 95 °C, and 45 cycles of 95 °C for 10 s, 60 °C for 10 s, and 72 °C for 10 s. A final melting program ranging from 65 °C to 95 °C at increments of 0.5 °C and acquiring the fluorescence after each step. The reaction mixture contained 1 μl of diluted cDNA, 10 μl of SYBR TB Green mix (TaKaRa, Dalian, China), 1 μl of each sense and anti-sense, and 7 μL of ddH_2_O in 20 μl of total reaction volume. To ensure repeatability of the experiments, each reaction was run in triplicate. A ten-fold dilution series of cDNA samples was used to generate a relative standard curve. The correlation coefficient (R^2^) and slope were obtained from the linear regression model created by the BioRad CFX96^™^ Real-Time PCR system. PCR amplification efficiency (E) was calculated according to the formula: E = (10^(−1/slope)^ − 1) × 100.

### Reference genes expression stability analysis

The expression stability of the selected reference genes was evaluated using four Microsoft Excel-based programs: geNorm [23], Normfinder [24], Bestkeeper [25], Delta Ct [26] and a comprehensive online tool RefFinder [27] (http://150.216.56.64/referencegene.php) according to the developer’s instructions. GeNorm was also used to calculate the pairwise variation (Vn/Vn+1 value) to determine the suitable number of reference genes required for accurate normalization.

### Validation of reference genes

The muscle fiber differentiation-related gene Myogenin (*MyoG*) was selected as the target for validating the two most stable reference genes, *UXT* and *RPL13A*. The reference genes that varied the most, particularly, *GAPDH*, was also used to normalize the expression of target gene. The primers sequences for *MyoG* are shown in Table 1. RT-qPCR was performed as described above. The 2^-ΔΔCt^ method was used to calculate the relative normalized expression in EDL and GB muscle [32]. Data were expressed as mean ± SD of three biological replicates for each muscle. The differences on the *MyoG* expression levels were evaluated by Student’s t test using IBM SPSS Statistics 23.0 (SPSS Inc., Armonk, NY, USA).

### Measurement of muscle fiber characteristics

#### MyHC expression

Gene-specific primers for the quantification of mRNA expression of *MyHC I* and *MyHC IIB* gene were designed using Primer 5.0 software (S2 Table). RT-qPCR was performed as described above. The relative expression of *MyHC I* and *MyHC IIB* mRNA were calculated via the 2^-ΔΔCt^ method [32].

#### Mitochondrial DNA (mtDNA) copy number

Genomic DNA was isolated using the animal tissue Genomic DNA Kit (ZDGSY, Beijing, China) following the manufacturer’s instructions. The copy number of mtDNA per cell was determined using RT-qPCR according to the previous method [33,34]. ATP synthase subunit a (*ATP6*), cytochrome C oxidase I (*COX1*) and NADH dehydrogenase, subunit 1 (*ND1*) and glucagon (*GCG*) gene were selected to calculate the number of mtDNA copies per cell. All the primers used are described in S2 Table. RT-qPCR was performed as described above. The following modified equation was used to determine the mtDNA copy numbers of per cell: mtDNA copy numbers = [(No. of copies of the mtDNA gene)/(No. of copies of GCG)/2].

## Results

### Selection of candidate reference genes

To select suitable reference genes for the ten skeletal muscles of yak, we chose 14 candidate reference genes (*GAPDH*, *ACTB*, *UXT*, *TPB*, *YWHAZ*, *RPL13A*, *SDHA*, *RPS15*, *HPRT1*, *PPIA*, *HMBS*, *MRPL39*, *B2M* and *PPP1R11*) based on previous literatures. Candidate reference genes were distributed among different chromosomes that belong to different function classes to minimize the risk of coregulation.

### Primer specificity and amplification efficiency of candidate genes

Gene name, primer pair sequences, amplicon size, PCR efficiency (E), and correlation coefficients (R^2^) of the reference genes are provided in Table 1. The sizes of all PCR products were between 79–198 bp, and melting temperatures ranged from 84 °C to 88 °C. All PCR assays produced a specific amplification of the expected size, which exhibited a single sharp peak in the melting curve (S1 Fig). The amplification efficiencies of all the primers for the reference genes ranged from 93–109%, with R^2^ varied between 0.9921 and 1.0000 (Table 1).

### Expression levels of candidate reference genes

**Fig 1:**
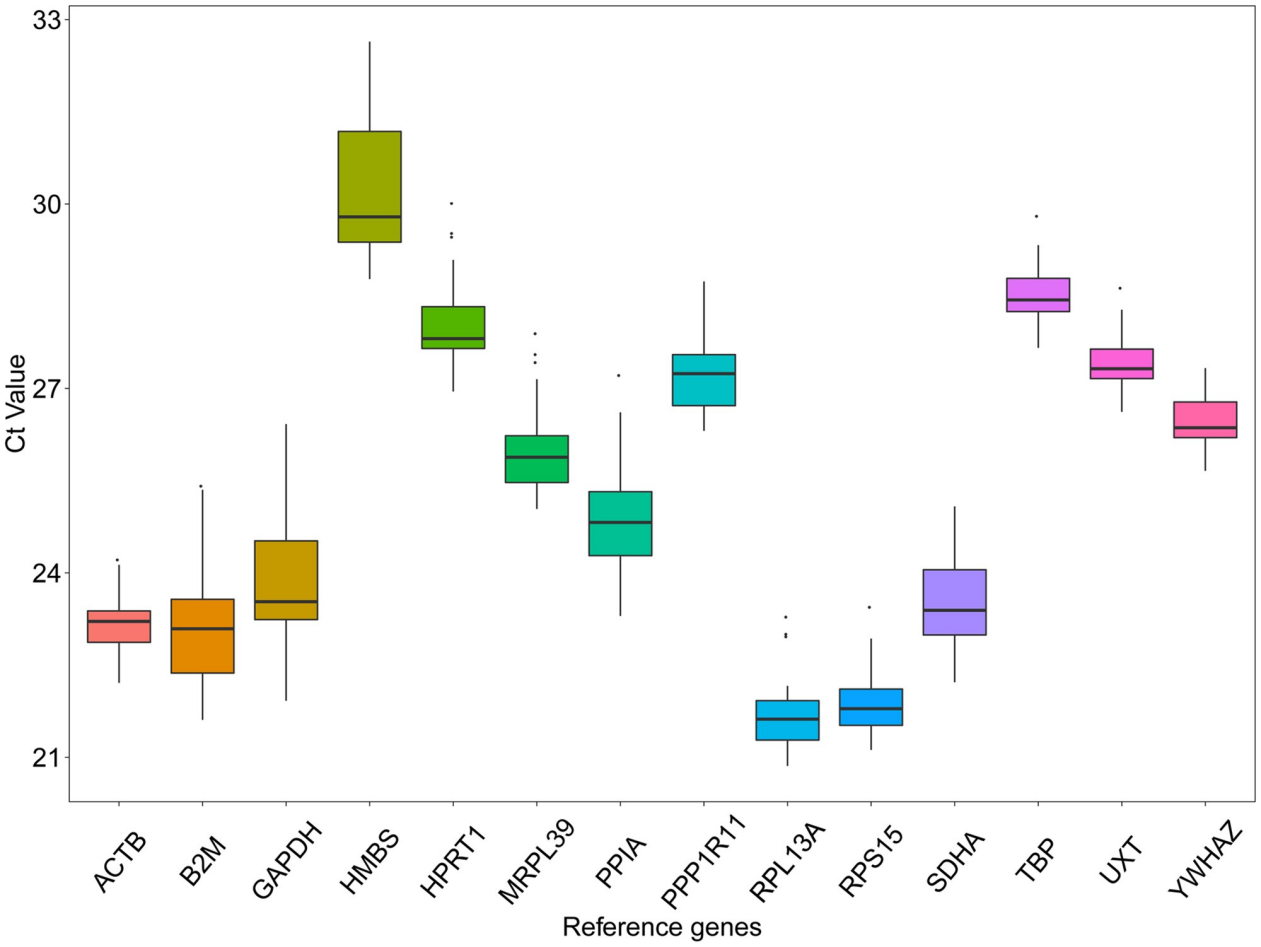
Ct values (expression levels) of 14 candidate reference genes in all tested samples. Each box indicates the 25th and 75th percentiles. Whiskers caps correspond to the maximum and minimum values. A line within the boxes depicts the median.

The Ct values of 14 candidate reference genes in all of the tested samples were shown in Fig 1. The mean Ct values of the 14 candidate reference genes exhibited relatively different variation in the ten different muscle tissues (S3 Table). Mean Ct values of candidate reference genes were between 20.86 to 32.64. The smaller the Ct value, the higher gene expression, and vice versa. The *RPL13A* gene had the lowest mean Ct value (21.73), corresponding to its highest level, while *HMBS* was the lowest expressed gene with a mean Ct value of 30.24.

### Stability of the reference genes

In this study, the stability of expression of the reference gene was analyzed by GeNorm, NormFinder, BestKeeper and Delta-Ct program. Then, the genes were re-ranked based on geometric mean by using the RefFinder online tool. The results of each program are provided in Table 2.

**Table 2:** Stability of reference genes in ten different muscle tissues of yak.

Rank	GeNorm		NormFinder		BestKeeper		Delta Ct		ReFinder	
**1**	RPL13A	0.242	PPP1R11	0.255	UXT	0.351	UXT	0.579	UXT	1.189
**2**	UXT	0.242	UXT	0.293	YWHAZ	0.352	RPS15	0.596	RPL13A	3.130
**3**	RPS15	0.267	HPRT1	0.307	ACTB	0.369	PPP1R11	0.597	PPP1R11	3.350
**4**	YWHAZ	0.328	RPL13A	0.315	TBP	0.381	RPL13A	0.600	RPS15	3.500
**5**	ACTB	0.366	RPS15	0.321	RPS15	0.390	HPRT1	0.619	YWHAZ	4.450
**6**	PPP1R11	0.413	ACTB	0.371	RPL13A	0.441	ACTB	0.629	ACTB	4.821
**7**	HPRT1	0.438	YWHAZ	0.421	PPP1R11	0.523	YWHAZ	0.649	HPRT1	5.692
**8**	TBP	0.469	SDHA	0.468	MRPL39	0.569	TBP	0.707	TBP	6.928
**9**	MRPL39	0.496	TBP	0.481	SDHA	0.585	SDHA	0.707	SDHA	8.972
**10**	SDHA	0.520	MRPL39	0.488	HPRT1	0.586	MRPL39	0.709	MRPL39	9.212
**11**	PPIA	0.565	PPIA	0.657	PPIA	0.620	PPIA	0.827	PPIA	11.000
**12**	HMBS	0.607	HMBS	0.700	B2M	0.766	HMBS	0.866	HMBS	12.243
**13**	B2M	0.644	B2M	0.739	HMBS	0.925	B2M	0.895	B2M	12.742
**14**	GAPDH	0.730	GAPDH	1.159	GAPDH	0.988	GAPDH	1.247	GAPDH	14.000

#### GeNorm analysis

The GeNorm program evaluated the stability of reference genes based on expression stability value (M). Genes with the lower the M value have the higher the expression stability. All of the analyzed reference genes had an M-value below the recommended cut-off value of 1.5, indicating that each reference gene has a relative stability across the tested samples (Table 2). According to the GeNorm result, the *UXT* and *RPL13A* had the highest stability, with the lowest M-value (0.242). In contrast, *GAPDH* exhibited the least stable (M value of 0.730) (Table 2).

**Fig 2:**
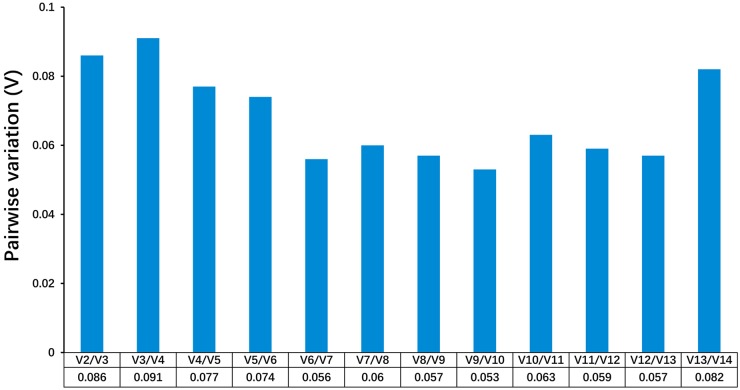
Pairwise variation (V) analyses in all tested samples. The V cutoff value was set to 0.15, below which the inclusion of an additional reference gene would not have a beneficial effect on the normalization.

GeNorm was also used to calculate the pairwise variation (Vn/Vn+1) between sequential normalization factors (NF) to determine the minimum number of genes necessary for normalization. A cutoff value of 0.15 is the recommended threshold to identify the benefit of additional reference gene for the normalization [23]. As evident in Fig 2, pairwise variations V2/3 in ten skeletal muscles were less than 0.15, which indicates that two reference genes were suitable for gene expression analysis (Table 2).

#### NormFinder analysis

According to the NormFinder program, genes with lower average expression stability values present with more stable expression. NormFinder ranking indicated that *PPP1R11* and *UXT* had the most stable expression with stability values of 0.255 and 0.293, while *B2M* (0.739) and *GAPDH* (1.159) were ranked the least stable genes with the higher stability values (Table 2).

#### BestKeeper analysis

The BestKeeper calculates the stability of the candidate reference genes on the basis of standard deviation (SD) of Ct values and the correlation coefficient (r) of expression among the reference genes. Determining the gene stability based on the SD value is the more conservative approach [35]. A gene with the lowest SD value is ranked as the most stable reference gene, while a gene with a SD above 1 is not a stable reference gene. BestKeeper indicated that SD values of all reference genes were less than 1. *UXT* and *YWHAZ* had the lowest SD value (0.351) and thus were the most stable genes in the tissue, while *HMBS* (0.925) and *GAPDH* (0.988) were the least expressed genes (Table 2).

#### The Delta Ct analysis

The Delta Ct program ranks the most stable reference genes by comparing relative expression value of tested reference genes in pairs in all of the samples. Genes with lower SD values represent the higher expression stability. The ranking of reference genes was similar to what was obtained using the GeNorm algorithm. *UXT* was the most stable reference gene in ten skeletal muscles with the lowest SD value (0.579), whereas *GAPDH* (1.247) had the lowest stability (Table 2).

#### RefFinder analysis

To avoid the divergent stability rankings of different software, the online RefFinder tool was also used to construct a comprehensive ranking of the most stable candidate reference genes. Reference genes with the lowest geometric mean are recommended by RefFinder. According to the recommended comprehensive ranking, *UXT* and *RPL13A* were the most suitable genes for endogenous controls because they had the lowest geometric mean of ranking value, indicating they were the most stable (Table 2).

### Reference gene validation

**Fig 3:**
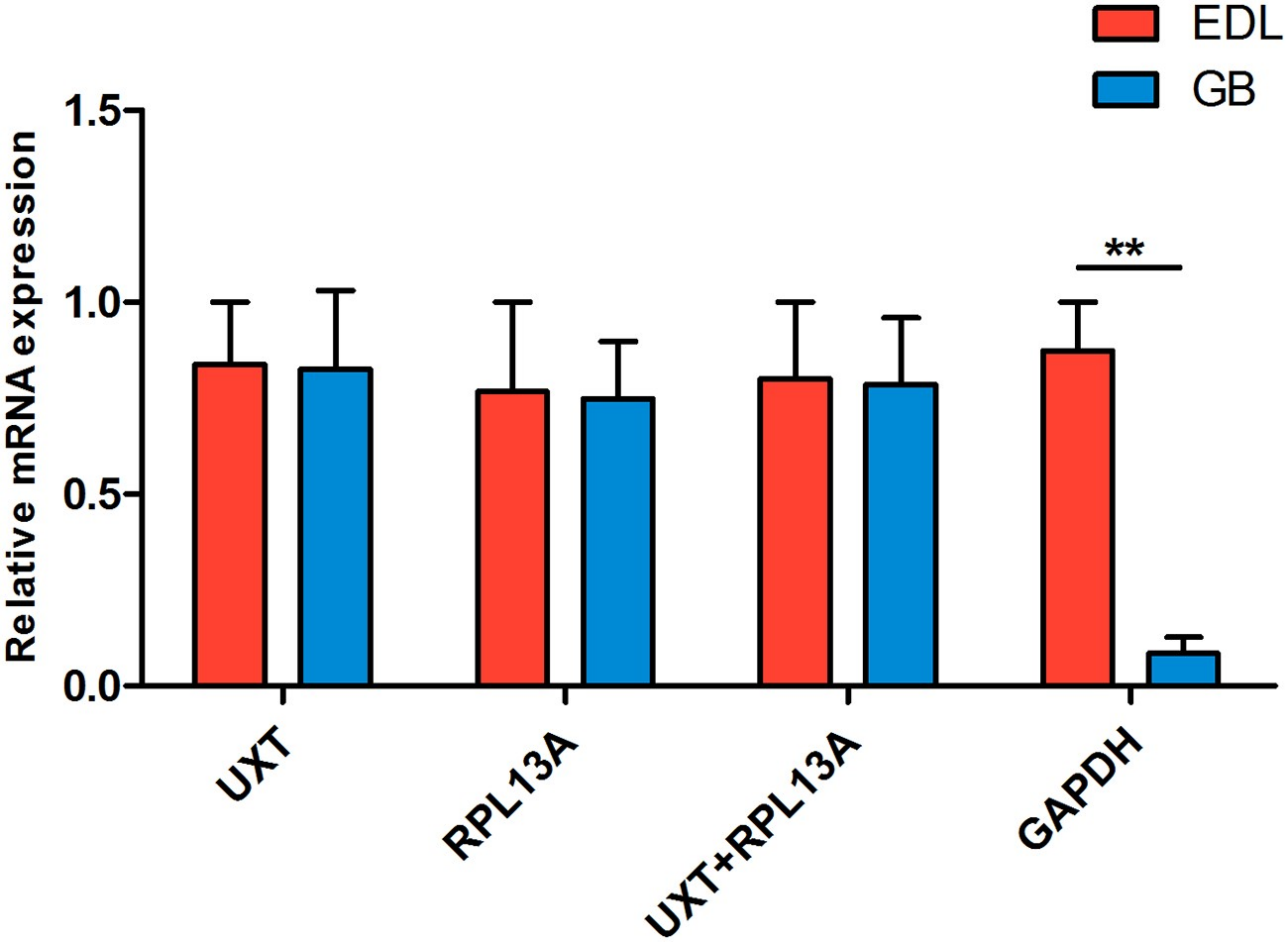
The normalized expression of the *MyoG* mRNA in EDL and GB muscle of yak. The expression levels were normalized using *UXT*, *RPL13A* or *GAPDH* as reference genes individually and with the mean of *UXT*+ *RPL13A*. **P < 0.01.

The relative expression of *MyoG* was used to validate the stability of the best-ranked candidate reference genes. When normalized using the stable reference genes *UXT* and *RPL13A* either alone or in combination, there was no significant difference in *MyoG* expression between EDL and GB muscle. On the contrary, when the least stable reference gene *GAPDH* was used for normalization, the expression of *MyoG* in EDL muscle was significantly higher than that in GB muscle (Fig 3).

### The characteristics of skeletal muscle fiber types

**Fig 4:**
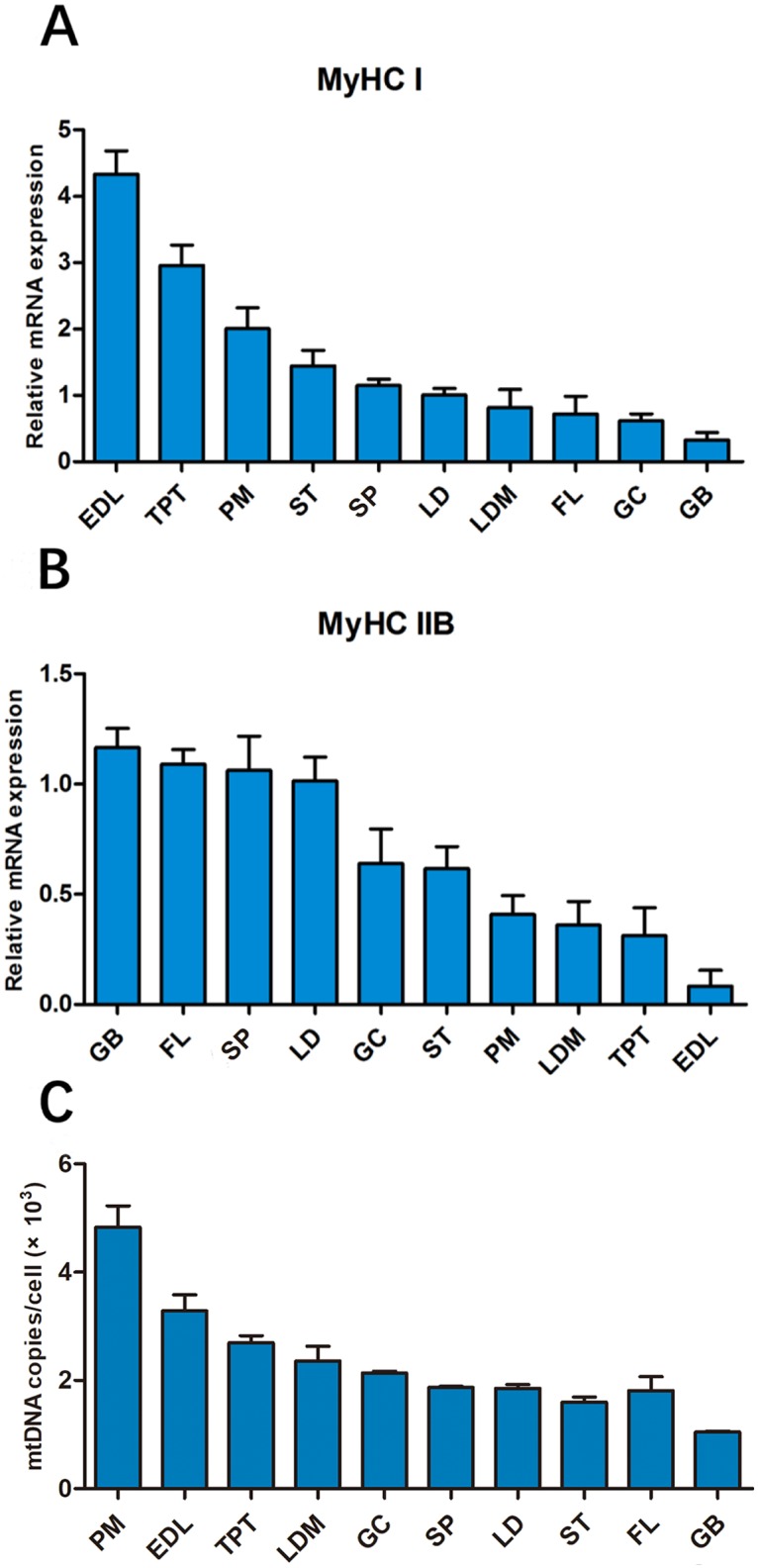
The characterization of ten different skeletal muscles. (A and B) Normalized expression of *MyHC I* and *MyHC IIB* in ten different muscle tissues, (C) MtDNA copies per cell in ten different muscle tissues.

The myofiber characteristics of ten different muscles from yak are shown in Fig 4. The muscle had higher percentages of type I fibers and lower percentages of type IIb fibers, and vice versa. Among these muscles, EDL, TPT and PM muscle had the higher expression of *MyHC I* mRNA and copy number of mtDNA per cell, indicating that their oxidative capacity was higher compared with those of other skeletal muscles. In contrast, GB muscle had the highest expression of *MyHC IIB* mRNA and lowest copy number of mtDNA per cell, suggesting it to be more proficient in anaerobic glycolytic metabolism.

## Discussion

Improving meat quality is part of the main breeding objectives for domestic animal production. Previous studies have shown that muscle fiber composition directly influences the eating quality of meat, as they are the basic component of skeletal muscles [36]. One of the important aspects in the study of muscle fiber composition is the expression analysis of *MyHC* genes by RT-qPCR. However, RT-qPCR data can be easily influenced by the stability of the reference genes chosen for RT-qPCR data normalization. Using unsuitable reference genes can lead to deviations in expression normalization and thus generate incorrect results [37]. The optimal reference gene should be constantly expressed in all types of organisms throughout the different development stages [18]. In reality, there is not an ideal set of genes that are consistent in all organisms. Therefore, it is necessary to select and validate suitable reference genes in different cases for expression analysis prior to RT-qPCR. In this study, 14 reference genes of yak were selected, and their expression was evaluated using a multi-algorithm analysis in ten different muscles of yak. To the best of our knowledge, this is the first study to select and validate the reference gene for different muscle tissues of yak.

To evaluate the gene expression stability of the selected genes among different skeletal muscles, we used four programs (GeNorm, NormFinder, BestKeeper and Delta CT) to obtain rankings of 14 candidate reference genes. GeNorm uses average pairwise variation of each reference gene to calculate its gene-stability value (M) [23]. BestKeeper estimates gene expression stability on the basis of standard deviation (SD) The NormFinder calculates expression stability according to the estimated intragroup and intergroup variation [24]. BestKeeper estimates gene expression stability on the basis of standard deviation (SD) [25]. The Delta-Ct method compares the relative expression of reference gene in pairs to rank the stability based on reproducibility of gene expression variation. [26]. In previous studies, as in ours, some discrepancies of stability rankings for reference genes were presented using different programs. The Best Keeper and Delta Ct methods identified *UXT* as the most stable reference gene in different skeletal muscles, but NormFinder recommended *PPP1R11* as the most stable reference gene. GeNorm recommended *RPL13A* and *UXT* as the most stable genes, respectively. These divergent stability rankings for the reference genes may be attributed to the distinct mathematical models of each algorithm [38]. To address this problem, the web-based tool RefFinder was used to select the most stable reference genes for accurate normalization. It is necessary to unify the inconsistent results in previous scientific papers [18].

According to the literature, *GAPDH*, which is involved in primary metabolism and other cellular processes, has been mostly used to normalize the quantitative expression in yak [39,40]. It was assumed that the expression *GAPDH* was quite stable in many other mammalian species. However, several studies have shown that *GAPDH* is not an ideal reference gene in developing skeletal muscle. For example, *GAPDH* was considered as the optimal reference gene in human muscle [41], however *GAPDH* exhibited poor performance in the muscle tissues in several animals including mouse, cattle, goat, and pig [38,42–44]. Our data showed that *GAPDH* was identified as the least stable reference gene with any of the algorithms. These results highlight the fact that the most commonly used reference genes may vary considerably under some experiments and selection of the best reference genes is necessary under specific experimental conditions and species. The *ACTB* gene usually presents as a high stability gene, and it was considered as stable reference gene in skeletal muscle tissue of cattle and pig [43,45]. Li et al. investigated stabilities of ten reference genes, including *GAPDH* and *ACTB*, in six tissues of fetal yak. They found that *TBP* and *ACTB* were the two most stable, while *GAPDH* performed poorly [21]. However, our results showed that *ACTB* could be regarded as the third most stable gene based on the BestKeeper program while its ranking position indicated moderate stability in other three programs and RefFinder, this could be more applicable in various other tissue types other than the skeletal muscle of yak.

Previous studies have demonstrated that using at least two reference genes is sufficient to improve the accuracy and reliability of data normalization [17]. The GeNorm V values showed that all reference genes with a mean pairwise variation value <0.15, demonstrating that two reference genes were enough for gene expression analysis, and the addition of one more reference gene would not significantly improve accuracy [23]. In the present study, the comprehensive ranking of RefFinder analysis showed that *UXT* and *RPL13A* were the best reference genes that were expressed with the highest stability. UXT serves as a cofactor which regulates gene transcription, and it also plays an essential role in concert with the corepressor URI1 to regulate androgen receptor AR-mediated transcription [46]. *UXT* has previously been found suitable as a reference gene for cells and tissues of some ruminants, including taurine cattle, zebu, riverine buffalo, and goat [47–49]. Recent studies showed that *UXT* was also selected as a suitable reference gene in yak. During pregnancy and lactation stage, *MRPS15*, *RPS23*, and *UXT* were the most stable gene in yak mammary tissue [19]. Bai et al. investigated the stability of thirteen reference genes and found that *RPS9*, *PPP1R11*, *UXT*, and *MRPL39* were the most stable reference genes to normalize RT-qPCR data from milk somatic cells of yak [20]. *RPL13A* encodes one of the protein components of the large 60S ribosomal subunit, and it has an important role in regulation of translation of specific mRNAs as part of a non-ribosomal complex [50,51]. Notably, some ribosomal protein genes (*RPL13A*, *RPS5*, *RPL32* and *RPS19*) have been highly regarded as suitable reference gene in canine metencephalon and body tissues [52,53]. *RPL13A* was the reference gene in 11 distinct brain regions of normal humans [54] and in mouse lung tissues [55]. Therefore, to obtain the reliable gene expression results from various skeletal muscle of yak, we selected *UXT* and *RPL13A* as the most appropriate pair of reference genes.

To validate the best selected reference genes, the relative expression level of the *MyoG* gene was measured in muscle samples from different muscle fiber types. MyoG is a muscle-specific transcription factor that belongs to myogenic regulatory factor family. It plays a role in muscle differentiation and cell cycle exit [56]. Significant differences were revealed in the expression patterns of the *MyoG* gene when it was normalized with the two most stable genes (*UXT* and *RPL13A*) compared to when it was normalized with one of the least stable genes (*GAPDH*). These results showed that selection of reliable reference genes is necessary to improve the accuracy of normalization results.

Many previous reports showed that MyHC I, MyHC IIA, MyHC IIX and MyHC IIB isforms are expressed respectively in muscle fibers, and that the MyHC IIB isform is not present in skeletal muscles of cattle and buffalo [57,58]. We used the RT-qPCR to perform *MyHC* isoform mRNA expression in ten different skeletal muscles of yak, and found that *MyHC IIB* is expressed in yak muscle. Wang also found that the MyHC IIB isoform protein was expressed in yak muscle [59]. These differences may be attributable to species variation. In this study, there were significant differences in muscle fiber characteristics among ten different muscles. Different metabolic capacity based on the copy number of mtDNA was consistent with the muscle fiber composition. These differences may be attributed to the different location of muscle and variable requirements for oxygen diffusion in muscle fiber [7]. PM muscle is a typical oxidative muscle fiber type. Our results showed that PM muscle had the higher type I fibers and mitochondrial content, which is consistent with measurements made by Lang et al [36]. Hwang et al. reported that LD muscle of Hanwoo (Korean native cattle) steers had a higher percentage of type IIB fibers [60]. In contrast, a moderate fat content was observed in LD muscle of yak in the present study. It is therefore reasonable to assume that the muscle types and species-specific molecular regulation network is due to species variation (taurine cattle vs. yak).

## Conclusions

To validate suitable reference genes for gene expression normalization in skeletal muscle tissue of yak, we selected 14 candidate reference genes using four systematic statistical algorithms (geNorm, NormFinder, Delat Ct, and BestKeeper). The obtained results were compared and ranked using RefFinder. Based on our comprehensive analysis, we identified *UXT* and *PRL13A* as the most stable reference genes for normalization of gene expression across different muscle tissues. We also examined the gene expression of muscle fiber in ten different skeletal muscles, providing a basis for possible selection towards improved meat quality of yak.

## Supporting information

S1 Fig Melting curves of ten genes of RT-qPCR. (TIF)

S1 Table Ct values of candidate reference genes in all muscle samples. (XLS)

S2 Table The nucleotide sequences of these 14 reference genes. (DOC)

S3 Table Primer sequences for muscle fiber composition related genes. (DOCX)
