# Supplementary figures and images for: Reference gene selection and myosin heavy chain (MyHC) isoform expression in muscle tissues of domestic yak (Bos grunniens)
Source: PLoS One. 2020 Feb 6;15(2):e0228493. doi: 10.1371/journal.pone.0228493 (PMC7004298; doi:10.1371/journal.pone.0228493)

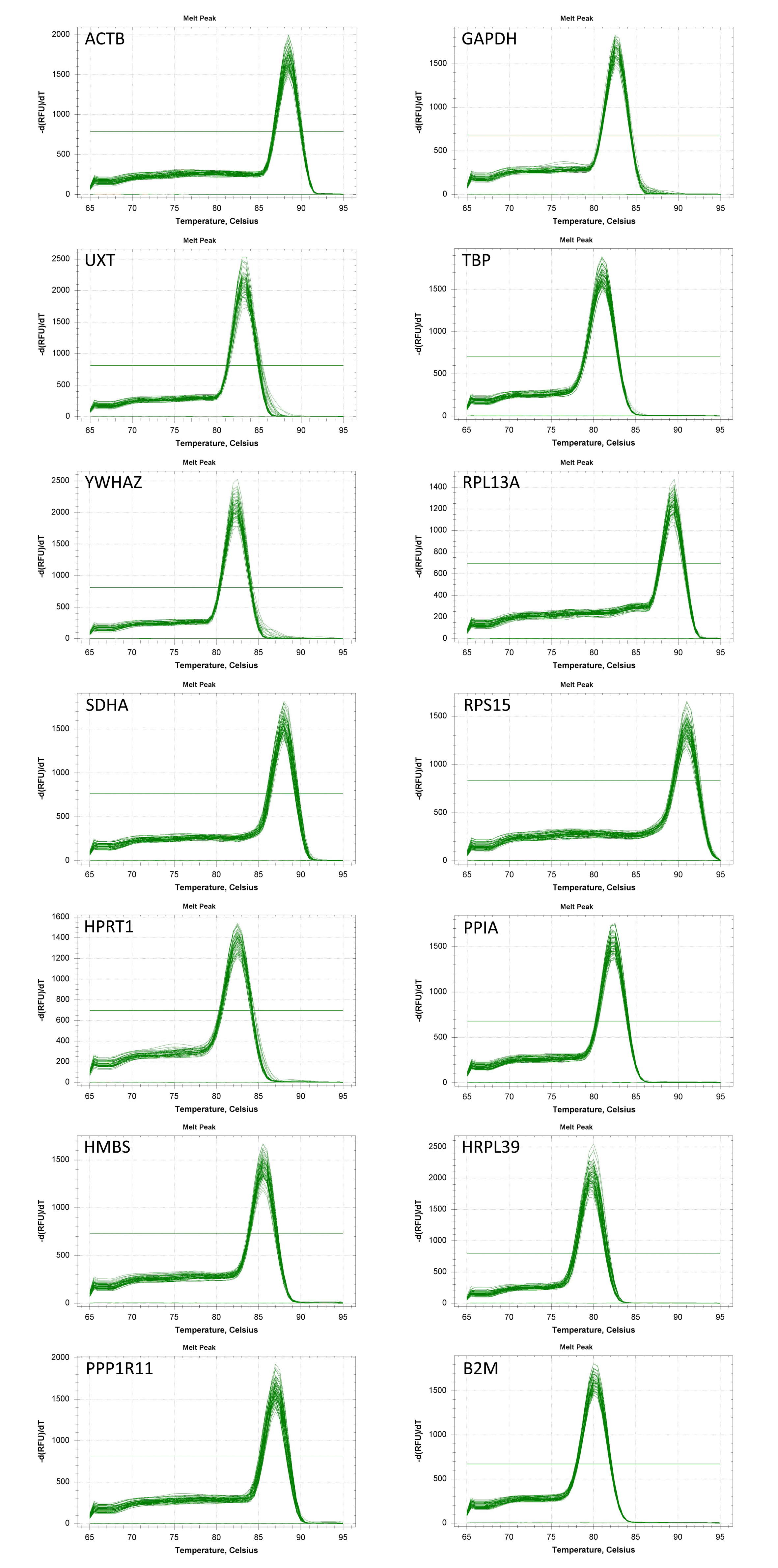

Supplement: S1 Fig — (TIF) [file pone.0228493.s001.tif]
