# Supplementary material for: Reference gene selection and myosin heavy chain (MyHC) isoform expression in muscle tissues of domestic yak (Bos grunniens)
Source: PLoS One. 2020 Feb 6;15(2):e0228493. doi: 10.1371/journal.pone.0228493 (PMC7004298; doi:10.1371/journal.pone.0228493)
Supplement: S2 Table — (DOC) [file pone.0228493.s003.doc]

**S2 Table. The nucleotide sequences of these 14 reference genes.**

| Genes | Nucleotide sequences |
| --- | --- |
| ACTB | TCCCTCATCTGGCCGCCCGCGCGCCGGCCCCCCCTAGCGGCTCGAAGGCGCGCTGCGCCGGAAGTGGGTAGGGCGGGGGCGGCCGCGCCCGACACCCTCCTACATCCCGGTTTACCCCCCGGTTGTCCCCACAGTTCGCCATGGATGATGATATTGCTGCGCTCGTGGTCGACAACGGCTCCGGCATGTGCAAGGCCGGCTTCGCGGGCGACGATGCTCCCCGGGCCGTCTTCCCGTCCATCGTGGGGCGCCCCCGGCACCAGGGCGTAATGGTGGGCATGGGCCAGAAGGACTCGTACGTGGGGGATGAGGCTCAGAGCAAGAGAGGCATCCTGACCCTCAAGTACCCCATTGAGCACGGCATCGTCACCAACTGGGACGACATGGAGAAGATCTGGCACCACACCTTCTACAACGAGCTCCGTGTGGCCCCTGAGGAGCACCCCGTGCTGCTGACCGAGGCCCCCCTGAACCCCAAGGCCAACCGTGAGAAGATGACCCAGATCATGTTCGAGACCTTCAACACCCCTGCCATGTACGTGGCCATCCAGGCTGTGCTGTCCCTGTATGCCTCTGGCCGCACCACCGGCATCGTGATGGACTCCGGTGACGGGGTCACCCACACGGTGCCCATCTACGAGGGGTACGCCCTTCCCCATGCCATCCTGCGTCTGGACCTGGCTGGCCGGGACCTGACGGACTACCTCATGAAGATCCTCACGGAGCGTGGCTACAGCTTCACCACCACGGCCGAGCGGGAAATCGTCCGTGACATCAAGGAGAAGCTCTGCTACGTGGCCCTGGACTTCGAGCAGGAGATGGCCACCGCGGCCTCCAGCTCCTCCCTGGAGAAGAGCTACGAGCTTCCTGACGGGCAGGTCATCACCATCGGCAATGAGCGGTTCCGCTGCCCTGAGGCTCTCTTCCAGCCTTCCTTCCTGGGCATGGAATCCTGCGGCATTCACGAAACTACCTTCAATTCCATCATGAAGTGTGACGTCGACATCCGCAAGGACCTCTACGCCAACACGGTGCTGTCCGGCGGGACCACCATGTACCCCGGCATCGCAGACAGGATGCAGAAAGAGATCACTGCCCTGGCACCCAGCACAATGAAGATCAAGATCATCGCGCCCCCTGAGCGCAAGTACTCCGTGTGGATTGGCGGCTCCATCCTGGCCTCGCTGTCCACCTTCCAGCAGATGTGGATCAGCAAGCAGGAGTACGATGAGTCCGGCCCCTCCATCGTCCACCGCAAATGCTTCTAGGCGGACTGTTAGCTGCGTTACACCCTTTTTCTTGACAAAACCTAACTTGCGCAGAAAACGAGATGAGATTGGCATGGCTTTATTTGTTTTTTTTTTTTGTCTTTTTTGATTTTTTTTTTTGTTTTTTTTGGCGCTTGACTCAGGATTTAAAAACTGGAACGGTGAAGGTGACAGCAGTCGGTTGGATCGAGCATTCCCAAAGTTCTACAGTGTGGCCGAGGACTTGATTGTACATGGTTTTGTTTTTTTTTTAATAGTCATTCCAAATATCGCGAAATGCATTGTTACAGGAAGTCCTTTGCCTTCCCAAAAGCCACCCCGCTTCTCTCTAAGGAGAAGGGGCCAGTCCTCGCCCGAGTCCACACAGGGGAGGTGATCGCTTTTGTGTAAATTATGTACTCCAAAACAAATTTTGTTTTTAATCTTCGCCTTAATACTTGTTTTTTTTGTTTGTTTTATTTTGAATGGACAGCCATCATGGACCCCTTTTTTTGTCCCTCAACTTGAGATGTATGAAGGCTTTTGGTCCCCTTGGGAGCGGGTTGAGGTGCGGAGGCAGTCAGGGCTTTCCTGTACACTGACTTGAGACCAGTTCAATAAAGTGCACACCTTAAACACACA |
| GAPDH | ATGGTGAAGGTCGGAGTGAACGGATTCGGCCGCATCGGGCGCCTGGTCACCAGGGCTGCTTTTAATTCTGGCAAAGTGGACATCGTCGCCATCAATGACCCCTTCATTGACCTTCACTACATGGTCTACATGTTCCAGTATGATTCCACCCACGGCAAGTTCAACGGCACAGTCAAGGCAGAGAACGGGAAGCTCGTCATCAATGGAAAGGCCATCACCATCTTCCAGGAGCGAGATCCTGCCAACATCAAGTGGGGTGATGCTGGTGCAGAGTATGTGGTGGAGTCCACTGGGGTCTTCACTACCATGGAGAAGGCTGGGGCTCACTTGAAGGGTGGCGCCAAGAGGGTCATCATCTCTGCACCTTCTGCCGATGCCCCCATGTTTGTGATGGGTGTGAACCACGAGAAGTATAACAACACCCTCAAGATTGTCAGCAATGCCTCCTGCACCACCAACTGCTTGGCCCCCCTGGCCAAGGTCATCCATGACCACTTTGGCATCGTGGAGGGACTTATGACCACCGTCCACGCCATCACTGCCACCCAGAAGACTGTGGATGGCCCCTCCGGGAAGCTGTGGCGTGACGGCCGAGGGGCTGCCCAGAATATCATCCCTGCTTCTACTGGCGCTGCCAAGGCCGTGGGCAAGGTCATCCCTGAGCTCAACGGGAAGCTCACTGGCATGGCCTTCCGCGTCCCCACTCCCAACGTGTCTGTTGTGGATCTGACCTGCCGCCTGGAGAAACCTGCCAAGTATGATGAGATCAAGAAGGTGGTGAAGCAGGCGTCAGAGGGCCCCCTCAAGGGCATTCTAGGCTACACTGAGGACCAGGTTGTCTCCTGCGACTTCAACAGCGACACTCACTCTTCTACCTTCGATGCTGGGGCTGGCATTGCCCTCAACGACCACTTTGTCAAGCTCATTTCCTGGTACGACAATGAATTCGGCTACAGCAACAGGGTGGTGGACCTCATGGTCCACATGGCCTCCAAGGAGTAAGGTCCCTGGACCCCCAGCCCCAGCAGGAGCACGAGAGGAAGACTTCCTCAGCTGCTGGGGAGTCCTGCCCCATCTCCGCCACACTGAGAATCTCCTGACTTCCACATGTTTCCATCTCTAAGGCCCTGAGGAAGGGGAGGGGCTTAGGGAGCCCTGCCTTGTGTACCATCAATAAAAGTACCCTATACCCAG |
| UXT | CAGATTCATAATCTTTTCTTGCCCGACTGCTTCTGAAGCTCTGACTTTTAAATACCCAAAGCTCTCTGTGTGTCCCGCCTCCTCCGGAACCCGCCAAATCTCATCAAACCCCACCCCCAGCAACAGTGCACTATCCCCATAGGCTTGTTGCGCAGTCCAGGTTCCCACGCTCTGCAGCCTTCCTTAATCAGCGGATTTCTTAAAGCCCAGCCCTTTCCTTCTCTCCGGCGATTTTTTCCGTTCAGGACCGCCTCACCCCACCTGGTTATTTGGTTGGCCAGCGCCCCCGTCGTACGTCATTTACCCGCGCCACCCGGAAGCCGCGGTTCTTACTAACAGTTCTTTTTGCCGGCGGCTTTCAGGAGCCCATCATGGCGACGCCCCCTAAACGGCGGGCGGTGGAGGCCACGGCGGAGAAAGTGCTGCGCTACGAGGCTTTCATCTCTGACGTGCTGCAGCGGGACTTGCAAAAGGTCCTGGACCATCGTGACAAGGTATATGAGCAGCTGGCCAAATACCTTCAACTGAGAAATGTCATTGAGCGACTCCAGGAAGCTAATCACTCGGAGTTATATATGCAGGTGGATTTGGGCTGTAACTTCTTCGTTGACACAGTGGTCCCAGACACTTCACGGATCTATGTGGCCCTTGGATATGGTTTTTTCCTGGAGTTAACACTGGCAGAAGCTCTCAAGTTCATTGATCGTAAGAGCAATCTCCTCACAGAGCTCAGCGACAACCTCACCAAGGACTCCATGAATATCAAGGCCCATATCCACATGTTGCTAGAGGGGCTTAGAGAGCTACAAGGTCTGCAGAATTTCCCAGAGACTCAGCACTGACTTCTTCCTACCTCCCCTGACATTAAAGAGCCTGAATGTCTTTGA |
| TBP | GGAAGGGTCGCCGTGGCGGGCGCCTGGGCCGCTGGGTGCTGAACTTCGTTTTCTCCGACCGGTAGTCGTCTCTGCAGTGACTCCAGGAAATGGCAACCTACTCCAGTGTTCTTGCCTGGAGAATCCCAGGGACCGGGGAGCCTGGTGGGCTGCCGTCTCTGGGGTCGCACAGAGTCGGACATGACTGAAGCGAGTTAGCAGCAGCAGCAGCAGCGGCAGCAGCAGCAGCAGCACCGCTGTTTCTTGGTGTGCACGAAGATAACCCACAGAGCCGAGCAAGTCGCCAAGAATAGTGTGCTGGGGATGCTCTAGGAAAAGACTGAATGCTGAGGCAATTTCCAGTCCAGAGGTTTTTGATTTGCCAAGAAGGTGAACGTCATGGATCAGAACAACAGCCTCCCACCCTATGCCCAGGGCCTGGCCTCCCCTCAGGGTGCCATGACTCCTGGAATCCCTATCTTTAGTCCAATGATGCCTTACGGCACAGGACTGACTCCACAGCCTATTCAGAACACCAACAGTCTGTCTATTCTGGAGGAGCAGCAACGGCAGCAGCAGCAGCAGCAGCAACAGCAGCAGCAGCAGGCGGCAGTGGCAGCCGTCCAGCAGTCGACGTCCCAGCAGGCAACCCAGGGGCCCTCGGGCCAGACACCACAGCTCTTCCACTCACAGACTCTTACGACTGCACCCTTGCCGGGCACCCCTCCCTTGTATCCCTCCCCCATGACCCCCATGACCCCCATCACCCCTGCCACGCCAGCCTCAGAGAGCTCCGGGATCGTGCCGCAGCTGCAAAATATTGTATCCACAGTGAATCTTGGTTGTAAACTTGACCTAAAGACCATTGCACTTCGTGCCCGAAATGCTGAATATAATCCCAAGCGTTTTGCTGCTGTAATCATGAGAATAAGAGAGCCCCGCACCACTGCACTGATATTCAGTTCTGGGAAGATGGTGTGCACAGGAGCCAAGAGTGAAGAACAGTCTAGACTAGCAGCAAGAAAATACGCCAGAGTTGTACAGAAACTGGGTTTTCCAGCTAAGTTCTTGGACTTCAAGATTCAGAACATGGTGGGGAGCTGTGATGTGAAGTTCCCTATAAGGTTAGAAGGCCTTGTGCTTACCCACCAACAGTTCAGTAGTTATGAGCCAGAGTTATTTCCTGGTTTAATCTACAGAATGATCAAACCGAGAATTGTTCTCCTTATTTTTGTTTCTGGAAAAGTTGTATTAACAGGTGCTAAAGTCAGAGCAGAAATTTATGAAGCCTTTGAGAACATCTACCCTATTCTGAAGGGTTTCAGGAAGACAACGTAATGGCTGCTGTGTTCTCCTGCCTCCCCCACCCCCTTTTTTTTTTTTAAACCAATCAGTTTTGGTACACTCAGTGGTGTTGTGGACGGCCCCACGGGGTAGAGGATGGGCGTCTGGTTGTAGGGTGCGGCACCAGGTGAAGCGCCCTTCCACGCGCACCCCCGTGGGGTGCCGGAAGGGGTGTTGTTTCTGCACAGAGAACACCAGAGCGTCACTGTAAGTGGCTCAAACTGTGCTGCTATACGGGCAGTGCTGCCTGTTATTTATATTTAGATTTTAAACACTTACTGCTGTTGACAAGTTGGTTTAAGGGACAAAACTAAGTGTTAAAGCCACCTCAACAATTGATTGGACTTTATTTTGTTTAATTTCTTCCCCATAAACCACAGTTTTTATATTTCTACCAGAAAAGTAAAACTGTTTTTTAAAAAGTGTTGTTTTCTAATTTGTAACTCCTGAGGGTTATTTTTGTGCCAGACACATTCCGCCTTTTCAGTATTGCAGGACAGAATAGAAATGTATTAATGAAAACAAATGGCTGTACATATTTTTCTTCCTTCAGAGTACTCTGTACAATAAATGCTGTTTATAAAAGTGTTAGATTGATGTTGTAAATGAAACTTTGTAAGATTCATGTGATCATACTGTTAAAAAATTGTATTTTAGATATAATGCCTGAAACCA |
| YWHAZ | GCGGCTGTAGCCTGTGAGCAGCGAGATCCAGGGACAGAGTCTCAGCCTCGCCGCTGCCGCCCAGAGACTGCTGAGCCCCGTCCGTCCGCCGCCACCTACTCCGGACACAGAACATCCAGTCATGGATAAAAACGAGCTGGTACAGAAGGCCAAACTGGCCGAGCAGGCTGAGCGATATGATGACATGGCAGCCTGCATGAAGTCTGTAACTGAGCAAGGAGCTGAATTATCCAATGAGGAGAGGAATCTTCTCTCAGTTGCTTATAAAAATGTTGTAGGAGCCCGTAGGTCATCTTGGAGGGTCGTCTCCAGTATTGAGCAAAAGACGGAAGGTGCTGAGAAAAAACAGCAGATGGCTCGAGAATACAGAGAGAAAATAGAGACCGAGCTAAGAGATATCTGCAATGATGTACTGTCTCTTTTGGAAAAGTTCTTGATCCCCAACGCTTCACAAGCAGAGAGCAAAGTCTTCTATTTGAAAATGAAAGGAGACTACTACCGCTACTTGGCTGAGGTTGCAGCTGGTGATGACAAGAAAGGGATTGTGGATCAGTCACAGCAAGCATACCAAGAAGCTTTTGAAATCAGCAAAAAGGAAATGCAACCAACACATCCTATCAGACTGGGTCTGGCCCTTAACTTCTCTGTGTTCTATTATGAGATTCTGAACTCCCCTGAGAAAGCCTGCTCTCTTGCAAAGACAGCATTTGATGAAGCCATTGCTGAACTTGATACATTAAGTGAAGAGTCATACAAAGACAGCACGCTAATAATGCAGTTACTGAGAGATAACTTGACATTGTGGACATCGGATACCCAAGGAGACGAAGCTGAAGCAGGAGAAGGAGGGGAAAATTAACCTGCCTTCCAACTTTTGTCTGCCTCATTCTAAAATTTACACAGTAGACCATTTGTCAT |
| RPL13A | TCCCAGGCAGCTGCCGAAGATGGCGGAGGGGCAGGTCCTGGTGCTCGATGGCCGAGGCCATCTCCTGGGCCGCCTGGCGGCCATTGTGGCCAAGCAGGTGCTTCTGGTGAAATACCTGGCCTTTCTCCGCAAGCGGATGAACACCAACCCCTCCCGTGGCCCCTACCACTTCCGAGCCCCCAGCCGCATCTTCTGGCGGACAGTGCGAGGCATGCTGCCCCACAAGACCAAGCGGGGCCAGGCTGCTCTGGAGCGCCTCAAGGTGTTTGATGGGATCCCACCACCCTATGACAAGAAAAAGCGAATGGTGGTTCCTGCTGCCCTCAAGGTTGTGCGTCTGAAGCCTACTCGCAAGTTTGCCTACCTAGGGCGCCTGGCTCATGAGGTTGGCTGGAAGTACCAGGCAGTAACGGCCACCCTGGAGGAGAAGAGAAAGGAGAAGGCCAAGATCCACTATCGGAAAAAGAAGCAGCTCATGAGGCTACGGAAGCAGGCCGAAAAGAACATCGAGAAGAAAATTGGCAAATTCACAGAGGTCCTCAAGACTCATGGATTCCTAGTCTGAGCCAAATAAAATTGACTGTTTATTCTTCATGCTTGGCCTGGCCTGCCCTTCCTCCATCGCCACCCTAGGATGTGGGGGCCCCCAGGGGCTGCCGTGCACGTGCCACAGGCAGACGGGGTATAGCCGAGGGGCATTAGTCAGTGTAGGCGGGTCCAGGGGCTGTGCAGGCATCGTTGGTCTGCAGCCTGTTTGTTCGTGAGACCTTTAAGACATAAACAGTTGGAACAACCAGTCCAGTTGCAGTATTGGCAGACGTGAGCTGGAAGACTATTGGAAAGGATTGGCATGACTTAAAGGGAGTCTGTTCATGCTTCCCACGTGGTCGGATACTATACTCTGCAGCTGTTAGGATGTGAGAGAATGCCCAAAAAGTATCTCTGGAAGAGAAGTTCCAGCCAAGGGATCACAGGCATCGCCCTCGTGTCTTCCCTATATTTTGTGATCCAAAACCAATAAATTATTTTGTTAAAGAAA |
| SDHA | ATGAAGGGCCCGCCCCGGGGCGGCCTGCTCTGGAGCCCCACTGCCCACTCCTCACTGTCAGCGCCTCCTGCAAAGTGGCCGGCAGCATGGCAGACAGGAACCCGCAGTTTTCACTTCACCGTTGATGGCAATAAGAGGTCGTCCGCTAAAGTTTCAGATGCGATTTCTGCGCAGTACCCAGTTGTGGACCATGAGTTTGATGCCGTGGTGGTTGGTGCAGGGGGGGCAGGCCTGCGGGCCGCGTTCGGCCTCTCTGAAGCAGGTTTCAACACGGCCTGCATCACGAAGCTCTTCCCCACCAGATCCCACACTGTCGCGGCCCAGGGAGGGATCAATGCCGCCCTGGGGAACATGGAGGAGGACAACTGGCGGTGGCACTTCTACGACACCGTGAAAGGCTCCGACTGGCTGGGGGACCAGGACGCCATCCACTACATGACGGAGCAGGCCCCCGCCTCCGTGGTGGAGCTGGAGAATTACGGCATGCCCTTTAGCAGAACTGAAGATGGCAAGATCTACCAGCGTGCCTTTGGCGGACAGAGCCTCAAGTTCGGGAAGGGTGGGCAGGCTCACCGCTGCTGCTGCGTGGCCGACCGCACGGGCCACTCGCTGCTGCACACGTTGTATGGAAGGTCTCTGCGCTATGACACCAGCTACTTTGTGGAGTACTTTGCTCTGGACCTCCTGATGGAGAGCGGGGAGTGCCGCGGTGTGATCGCTCTGTGCATAGAAGACGGGTCCATCCACCGCATCAGGGCCAGGAACACTGTCATCGCCACCGGAGGCTACGGGCGCACCTACTTCAGCTGCACGTCCGCCCACACCAGCACCGGGGACGGCACTGCCATGGTGACCAGGGCTGGCCTGCCCTGCCAGGACCTGGAGTTCGTGCAGTTCCACCCCACAGGCATATATGGCGCTGGCTGTCTCATCACAGAGGGCTGTCGTGGAGAGGGGGGCATCCTCATCAACAGCCAGGGTGAGAGGTTCATGGAGCGCTACGCCCCCGTCGCTAAGGACCTGGCGTCCAGGGATGTCGTGTCCCGGTCCATGACCCTGGAAATCCGCGAGGGAAGAGGCTGTGGGCCCGAGAAGGACCACGTGTACCTGCAGCTGCACCACCTGCCCCCGGCGCAGCTGGCTATGCGCCTGCCTGGCATCTCAGAGACGGCCATGATCTTCGCGGGCGTGGACGTCACCAAGGAGCCCATCCCCGTGCTTCCCACCGTGCACTACAACATGGGTGGCATCCCCACCAACTACAAGGGGCAGGTTCTGAGGCACGTGAATGGCCAGGACCAGGTTGTGCCTGGCCTGTACGCATGCGGGGAGGCTGCCTGCGCCTCGGTGCATGGCGCCAACCGTCTGGGTGCAAACTCGCTCCTGGACCTGGTTGTCTTCGGCCGGGCGTGCGCCCTGAGCATCGCAGAGTCCTGCAGACCCGGAGATAAAGTTCCCTCGATTAAACCAAATGCTGGGGAAGAATCTGTCATGAATCTTGACAAATTGAGATTTGCCAATGGAAGCATAAGAACATCGGAACTGCGACTCAACATGCAGAAGTCGATGCAGAGCCATGCCGCGGTGTTCCGTGTGGGGAGTGTGCTGCAGGAAGGCTGTGAGAAGATCAGCAGCCTCTACGGAGACCTGCGGCATCTGAAGACGTTCGACAGGGGAATGGTCTGGAACACTGACCTGGTGGAGACCCTGGAGCTGCAGAACCTGATGCTTTGTGCTCTGCAGACCATCTACGGAGCGGAGGCCCGGAAGGAGTCGCGCGGCGCCCACGCCAGGGAGGACTTCAAGGAGAGGGTTGACGAGTACGATTACTCCAAGCCCATCCAGGGGCAGCAGAAGAAGCCCTTTGAGCAGCACTGGAGGAAGCACACGCTCTCCTACGTTGACATCAAGACTGGGAAGGTCACCCTGGAGTACAGACCTGTAATCGACAGAACTTTGAACGAGACTGACTGTGCCACTGTTCCCCCAGCCATCCGCTCCTACTGAGGAGACTGAGGCTTCCCTGCTCCTGTAATTATGTATAATAGCTCACGCCCGGGTTCAGGTCATCACCGCCTTCTTGATTGTGTCCTTGACAGACTGAGGAGGCCATCTGATAGAGATTCCTACCAGAGGCCAGTAACTTGCCAGTGAGTGTTCAGCCTGCCTTCACCCGTGCCTTCTTCTCGTGAATTAATAAAACTAGATGAAACATAAGTTA |
| RPS15 | ATGGGGGCGGAAGTGGAACAGAAGAAGAAGCGGACCTTCCGCAAGTTCACCTACCGCGGCGTAGACCTCGACCAGCTACTGGACATGTCCTATGAGCAACTGATGCAGCTATACAGCGCGCGCCAGCGACGGCGGATGCACNNNNNNNNNNGGAGGAAGCAGCACTCGCTGCTGAAGCGGCTGCGCAAGGCCAAGAAAGATGCGCCGCCCATGGAGAAGCCCGAGGTGGTGAAGACGCACCTGCGCGACATGATCATTCTACCCGAGATGGTGGGCAGCATGGTTGGCGTCTACAACGGCAAGACCTTCAACCAGGTGGAAATCAAGCCTGAGATGATCGGCCACTACCTAGGCGAGTTCTCCATCACTTACAAGCCCGTGAAGCATGGCCGGCCCGGTATCGGGGCCACCCATTCCTCCCGCTTCATCCCCCTCAAGTAACCTGCTGGCCAATAAAAGCAGAGATT |
| HPRT1 | ATGAAAAAAACCCAAGTCAAGATTAATGTGCAGAAAGTGGCAGGTCCCCAGTTCTTACAGTTGACCACTATTAGCGATGATGAACCAGGTTATGACCTAAATTTATTTTGTATACCCAATCATTATGCTGAGGATTTGGAGAAGGTGTTTATTCCTCATGGACTAATTATGGACAGGACCGAACGGCTGGCTCGAGATGTGATGAAGGAGATGGGTGGCCATCACATTGTGGCCCTCTGTGTGCTCAAGGGGGGCTATAAGTTCTTTGCCGACCTGTTGGATTACATCAAAGCACTGAACAGAAATAGTGACAAATCCATTCCTATGACTGTGGATTTTATCAGACTGAAGAGCTACTGTAACGACCAGTCAACAGGCGACATAAAAGTAATTGGTGGAGATGATCTCTCAACTTTAACTGGAAAGAATGTCTTGATTGTTGAAGATATAATTGACACTGGGAAGACAATGCAGACTTTGCTTGCCTTGGTCAAGAAGCATAAACCAAAGATGGTCAAGGTTGCGAGCTTGCTGATGAAAAGGACCCCTCGAAGTGTTGGATATAAACCAGACTTTGTTGGATTTGAAATTCCAGACAAGTTTGTTGTGGGATATGCCCTTGACTATAATGAATACTTCAGGGACTTGAATCACGTGTGTGTCATTAGCGAAACTGGAAAAGCAAAATACAAAGCCTAAGATGAGAGTCCGAGTTGAGTTTGGAAATACCTGGCGTCCCAGTGAAATCACCAGTGACATGATCCAATGTTCTTGTTCTGTGGCCAGCTGCTTAATAGAGCTTATTGCATGTATCTCCTAAGAATTTGATCTGTTTTGTATTTTAGAAATGTCAGTTGCTGCATTCCCGAACTCTTCATTTGCACTACGAGCCTAAAGACTATCAGTTCCCTTTGGGTAGATTGTTGTTTGACTTGTGAATGAAATAGCTCTTAAACCACACCACTATTGACTGGAAATATGGAAATTGTATGTGTAAGAAACATTTGAAGAGAAGAATATATTAGTTTTTTAATTGGTATTTTAACTTTTATATGTTCAGGAAAGAACAGAAGTGACTGAATATTGTTCCCTATGCTACTGTGTGCTTAGGAAAGTAGCGAGAGGCAGTCTCGTACCCACGACAGCACTTTGAGGCATTGTTGTGTCAGAGAGACCGCATGTCCCGCAAGTGTTTCAGTAGGTTTCGGTAGTATTAACTATATTTTCTTGCTTGTTCAGATCATTTCTGGTGAATCTTTGTCAACAGTTCCTTTTCAATACAGGTCAGTAAGTTCCAAAAACCTACCATTTTTGAATTCTTCAGTGTAAAAATCCTTCAAATAAAGGCTATCTCTTTAAAGGAAA |
| PPIA | CCCCCACGTGTTCCTTCGCGACCGCGGTCGACGGCGGGCCCGTGGGCCGCGTCTCTTTTGAGCTGTTTGCAGACAAAGTTCCAAAGACAGCAGAAAACTTTCGTGCTCTGAGCACTGGAGAGAAAGGATTTGGTTATAAAGGTTCCTGCTTTCACAGAATAATTCCGGGATTTATGTGCCAGGGTGGTGACTTCACACGCCATAATGGTACTGGTGGCAAGTCCATCTATGGCGAGAAATTTGATGATGAGAATTTCATTTTGAAGCATACAGGTCCTGGCATCTTGTCCATGGCAAATGCTGGCCCCAACACAAATGGTTCCCAGTTTTTCATCTGCACTGCCAAGACTGAGTGGTTGGATGGCAAGCACGTGGTCTTTGGCAAGGTGAAAGAGGGCATGAATATTGTGGAAGCCATGGAGCGCTTTGGGTCCAGGAATGGCAAGACCAGCAAGAAGATCACCATTGCTGACTGTGGACAAATCTAATAAATTTGACTTGTGTTTTACTTAAGCACCAGACCATTCCTTCTGTAGCCCAGGAGAGCACCCCTTCACCCCATGTGCTTGAAATATCCTATAATCTTTGTGCTCTTGCTACAGTTCTTTGGGTTCCATATTTTCCTTATCCCCCTCCAAGTTTAGCTGGATTGCAAAGTTAAATTTATGATTATGAAATAAAAACTAAACAATTATCTGTC |
| HMBS | GTGACGCAGTGCCTTAGCGGAGCCGGGAGTGTGTGGCTGCTGGAGAAGCTGGAGACCTGCGCGAGCCCAGTGTCCGGCCGGCAGGGGCCTTCGGCTTCCCCGACACCGGAGGACGTTGGCGGCAGCCCCGGGCCTAGCTGCCGAGCACAGCCATGTCTGGTAATGGCAACGCGGCCGCAACAGCGGAAGAAGACACCCCAAAGATGAGAGTGATTCGAGTGGGTACCCGCAAGAGCCAGCTGGCTCGCATACAGACGGACAGTGTGGTGGCAACGCTGAAAGCTTTATACCCAGGCCTGCAGTTTGAAATAATTGCTATGTCCACCACGGGGGACAAGATTCTTGATACCGCGCTCTCTAAGATTGGAGAGAAGAGCCTGTTTACCAAGGAGCTGGAGCATGCTTTGGAGAGGAATGAAGTGGACCTAGTTGTTCATTCGCTGAAGGACCTGCCCACGGTGCTTCCTCCTGGCTTCACCATTGGAGCTGTCTGCAAGCGGGAGAGCCCCTATGATGCTGTTGTCTTTCACCCAAAATTTGTTGGGAAGACTCTAGAAACCTTGCCAGAGAAGAGTGTGGTAGGAACTAGCTCCCTGCGGAGAGCAGCCCAGCTGCAGAGAAAGTTCCCACATCTGGAGTTCAAGAGTATTCGGGGAAACCTCAACACGCGACTGCGGAAGCTGGATGAGCTGCAGGAGTTCAGTGCCATCATCCTGGCCGCAGCTGGCCTGCAGCGCATGGGCTGGCAGAACCGGGTGGGGCAGATCCTGCACCCTGAGGAGTGCATGTATGCTGTGGGTCAGGAAGGAGGTTGCAGTGTGCCAGTGGCAGTGCATACAGCTATTAAGGATGGGCAACTGTACCTGACTGGAGGAGTCTGGAGTCTGAATGGTGCAGAGACCATGCAAGACACCATGCAAACCACCATCCATGTCCCTGTCCAGCATGAAGATGGCCCTGAAGATGATCCACAGCTGGTGGGCATCACTGCCCGGAACATTCCACGACAACCCCAGCTGGCTGCTGAGAACCTGGGCATCAGCCTGGCCACCTTGTTGCTGAACAAAGGAGCCAAGAACATCTTGGATGTTGCACGGCAGCTCAATGAAGCCCACTAATTGGTCTGTGGGGCACAGGTGCCTGCCTTGCTGGTCACCCAGTGCCTACATCCCAGCCCTCTGCTACCTGAGAAGTGACTACCCCCACAGGATTGAACTGCAGGGCAGAGACTTCCAGAGACTTGTCTCACCGAGGGGCCTTGCCTCCCCAGGTGGGGGCTTCCTCTCTAGAGAAAAAAAAAAATCTTAGTAATAAGCCACAGCCTTTGAATGTAACCAGTTCTACTAATAAACCAAATTTAAAGGTG |
| MRPL39 | ATGCGCGCTCGGAGTTTCCGGAGAAGAGGACGGGGAAGGACCTGCGCGCGGAGGTTCCTACCGCCGCGGAGGAGGCAATGGCGGTGGTGGTCCGGGGGCTGAGGCTCTGGCGGGCCGCCCCCAGCGGCGGGGCTGCCTGGAGGTCTGTGGTCACATCACCTGCCTCTCAGCTGTCACCGACCGAACTGATAGAAATGCAGAATGATCTCTTTAACAAAGAGAAAAACAGGCAGCTGTCGCTAACTCCCCGAACTGAGAAGATTGAAGTGAAACACGTTGGTAAAACTGACCCTGGCACTATCTTTGTGTTGAATAAAAACGTTTCCACTCCTTATAGCTGTGCCATGCATTTAAGTGAATGGTACTGCAGGAAGTCTATTCTGGCTCTTGTGGACGGACAGCCTTGGGACATGTATAAACCTTTGACCAAGTCCTGTGAAATTAAATTTCTAACTTTCAAAGATGATGATCCAGGAGAAGTCAATAAGGCTTATTGGCGTTCTTGTGCCATGATAATGGGCTGTGTGATAGAGAGGGCATTCAAAGAGGAATATGTGGTCCATTTGGTCAGAGCCCCAGAAGTTCCAGTAATCGCTGGAGCCTTCTGCTATGACGTTGTTTTGGATAAGAGACTTGATGAGTGGATGCCAACAAAAGAGAACCTGCGTTCTTTCACAAAAGATGCTCGTGCTTTACTTTATAAAGATCTTCCATTTGAAACACTGGAAGTTGAAGCAAAAGTGGCATTAGAAATATTTCAGCACAATAAGTACAAAGTAGATTTCATAGAAGAGAAGGCATCTCAGAACCCTGAGAGAATAGTCAAGCTACACAGATTTGGTGACTTCATTGATGTGAGTGAGGGCCCTCTTATTCCAAGAACAAGTATTTGTTTCCAGTATGAAGTGTCAGCGGTTCACAATCTTCAAGCCACCCAGTCGAGTCTTGTACGAAGATTCCAGGGTCTCTCTCTACCTGTGCACTTAAGAGCACATTTTACGATATGGAATAAATTATTGGAAAGGTCTCGGAAAATGGTAACTGAAGATCAAACTAAGCCTACAGAGAAGAGTGCATCTACCTAGTAACTTCCTCAAATTTAAATATGTATAGTAAATTAAAATAAAACTTTTCTATGTAGTGTTTGTGCATGCTTATAAAA |
| PPP1R11 | GAACGCGTCACACCCGGAAGTAGGGAGCCGGAAGTGGGGTTGGACAGGTTATCCCCAGGGGTGGGGCAGCGGAGGCCCAGGAGGAGGGGGAAAAAAGAAGGTGGAGGATCCTGGCTGCTAATCTGAATCGATACCGATTCTCTTAGACCTCAGAGACACAGAAAAGACAGAAGGGTGCCTCATCCCCTTTCCTCCGCTTCTCTCTCTCCTCAGCCTTAGCCATGGCGGAGGCAGGGGCCGGGCTGAGTGAGACCGTCACTGAGACAACGGTTACCGTGACAACGGAGCCCGAGAACCGGAGCCTAACCATCAAACTTCGGAAACGGAAGCCAGAGAAAAAGGTGGAATGGACGAGTGACACTGTGGACAATGAACACATGGGCCGCCGCTCATCAAAATGCTGCTGTATTTATGAGAAACCTCGGGCCTTTGGCGAGAGCTCCACAGAGAGTGATGACGAGGAAGAGGAGGGCTGTGGTCACACACACTGTGTGCGGGGCCACCGCAAAGGACGGCGTCATGCAACCCCGGGACCAAGCCCCACCAGCCCTCCCCAGCCTCCTGACCCCTCCCAGCCCCCTCCAGGGCCAATGCAGCACTAAATTCCTCGCTCCCCCACCATTCCTGTGTCTGTCTGGCCCTGAATGTATTCATGTGGCTACTCGGGGACTAAACCCACGATTTGATCCCTTCTCCAGCCCCCTCCTCCCCTCTCCTCTGCCTGACAGAGGGAAGAGGGAGAGGAAGGTGGACAGAGATCCTGGAATTCTGACTTGCTGCTATTCCAGAACCTAGGCTTCTGGGTTCCCCCAGCCCTCATTTCTCCTTACAATACCCAGCCTCCTCTCTCCAGGGATCCAGGCATCTTGATCCCAATCTTTTTCCTTTGTTCTCACTGCCAAACTGCCTGTCCTGGGATCCAGTTATCTTGGCCCCTTGCACTCTCTACTTGAGTTCCAAACAGCTAAATTGGGTTTCCAGCAGCCCCAGCTTTCACTGCCAGGGTCCTAGTCAGATTCCAGGCAATCTTGCTCCAGCTATGCTTGTTAATCCTGGCTTAGAGCTCTTCCACTTATGTATTTATGTCATCCTAACTCTTAGTCGTTGCCTGTGGGATGTGAGGTCTTCTGTGAGACCTCAGGGCTCCTAGCCCTTTCCCTTCTCTCCTGCCCACTTCCCCCAAGCCCTTAAGAGGAGTTAGGAGAGAGGGAGGTCTTTGTCCTTCTCACCTTTAATGAGAAATGGAAAAAAGAAATGGGCATGTCCTCTCTCCTCACCGTTCTCATGTGACTAGGGTTTCTGACAAAACTGGCTCCAAGACTAGTCACTTAGAGCCCACTATCTCCTCAGCCTTTGGTCTTCCAACTTAGGAGACAGATCCGACCCAGGGGCCTGGGTCCCTGGGAGAGGATGGAAAAGGGAGGGAGCCAAGAGATGCAATCTCACCCCTTCCTTCCAAGGCCTTGATGTCTCTCCCAGCCCAGATATCCAGCCCTCCCAGCCCTCCCCACCCCTTACTGGGATCTGGATATCATTTTCCAGGCCTTTTGCAGTGCACAGTTGCAGAGATCAGTCAAATCCATACCACCACTGAGATCTCATTTATTGCCACAGATGCACAAAATAAATAACCCAACATCACAAAA |
| B2M | ACTATAAAGGCGAGCGCCGAACCCAGCCGCTCACTCCGCCACCGCGCAGCTGCTGCAAGGATGGCTCGCTTCGTGGCCTTGGTCCTTCTCGGGCTGCTGTCGCTGTCTGGACTGGACGCCATCCAGCGTCCTCCAAAGATTCAAGTGTACTCAAGACACCCACCAGAAGATGGAAAGCCAAATTACCTGAACTGCTATGTGTATGGGTTCCATCCACCCCAGATTGAAATCGATTTGCTGAGGAATGGGGAGAAGATTAAATCGGAGCAGTCAGACCTGTCTTTCAGCAAGGACTGGTCTTTCTACCTGCTGTCCCACGCTGAGTTCACTCCCAACAGCAAGGATCAGTACAGCTGCCGAGTGAAACACGTTACTTTGGAACAACCCCGGATAGTTAAGTGGGATCGAGACCTGTAAGCAACACCATCAAGATTTGAACATTCTTCATTTGGTATAATATCTGGAAAATTCTGTTTCCCTGCTCTTTAATACTGATATGCTTTTATGCTTTATGCACATAATCAGAAGTCATATTCATGTTACCACAAATACCTTCTTTATAATTTTACCTTGGGTGCTACATGTCCATGTTTGACCTTCCTAGGCAGGTGTCTGCAGTGGAGGTGCTGGCATCTTAGAGGTGGGGAAGACAGTTTCAGATTTAACATCAACATCTGGGTGTCTTCTGCATACAAAACTGGGTAATAAGATACTTATGCACGTTAAAGTAAGCCTCCAATTTTGTAACTTTGGTTACAATTTTGGGAAAATTTTGGAAAGATAATTTTTAGAGTTATGGAAATTTGTTAATTTTTAGAATTATGGAGATTGTAGATGAAGCATCTTGCCATATAAAATTTATATGTTATCTCCTATATCTGTTAGAAACTTGGTTGTGCCTGGTTTCACTATATATGTTGTTTCACTAAAACAATGGTAAAACATTGTGTGCAGCTTCTCAAAGGAAGAACAGCTGACTTCTCAATGTCAGTGGGATCCAGAACATAGCATAGAAAGTCATCATGAATGTGATGAAACAACTTCCTACCTAAAATTCTCTATTCAATAAAAATCTTTTAAAAAAAACGACTGCATGATAAACATTATTAGATAACTAAGATTGCTGCACCTGTCAGATCCTCACTAAGCATCTAAATGATACATTTGAGGCAGAGGGAAGGCCTAGTGAGAGAAAAGGGAGAGTAATAGAAATGTATCAATTTTGTAATCTAAATATAAAATAGTAATGGTTGGTAAAGTGGTTAGAAAAATAAAACACAATAACCTAAGTGTAGAAAGAGTAAACAATGTTAAAGTATTTGATGGACTTTTTACCCTGAAGGGTAAAGTTACAGACTTTTTGGTAATTTAAATATGTACAATATATTTTTGGGCTGTCCTCAAAAAGAACATAGGCTGAATGTTTAATATCCAACTAGAAGATGGCATAGGAGCTTATGGAGTGGTAGTAATGTTTTTTTCCAATACTCAGTGAATTTATCATAAAAAACTCATAGCTGGGGAAAAAGGCAAATTTACTTAGACTTATAAAGGTTTTCTTAATATACATAAGCAGACACATGCACTCATTAATAGAAGTCTATTATAGGTATTTTACAGGTAAAATCAGAAATAAGGATCTTTCCTGAAGTTACACCATAGAACTTCCCTGGTGGCCCAGTGGCCAAGACTCTGTGCTCTCAGTGCCACAGTGAGAATTTGTACGCCTCAACCGAAGTCCCACATGCTGCAACTAAACACCAGAGCAGCTAAATAAGTAAATACTTTAAAAAAATAAAAAAAGATGCCCACTGCCCGCTTTGAGTGATGTTATACTAGAGGTCCCAGCAGCAAATCATGACAATGAAAAAAAGTAAAAAGAATAAATATTTGAAGAGAAGAAACAAAATTGTATTACTTTGATGCTATATATAGATTATATAGAAATGTCAATAAGATATAAAAGATAAATTCTCTTATTAATCCTAATAATAAATTTGCTGGACAAAAACA |
