# Supplementary material for: Reference gene selection and myosin heavy chain (MyHC) isoform expression in muscle tissues of domestic yak (Bos grunniens)
Source: PLoS One. 2020 Feb 6;15(2):e0228493. doi: 10.1371/journal.pone.0228493 (PMC7004298; doi:10.1371/journal.pone.0228493)
Supplement: S3 Table — (DOCX) [file pone.0228493.s004.docx]

| **Gene** | **Accession No.** | **Primer Sequence (5’-3’)** | **Size (bp)** | **Efficiency (%)** | **R^2^** |
| --- | --- | --- | --- | --- | --- |
| ND1 | AY684273.2 | F: TACGCACTAATCGGAGCCCTAC  R: CTCGGTTTGTTTCTGCTAATGTTG | 208 | 96.0 | 0.9992 |
| ATP6 | [AY325806.1](https://www.ncbi.nlm.nih.gov/entrez/viewer.fcgi?db=nucleotide&id=32765607) | F: CCTCGTAACTCTTATCGTAC  R: AGGTTTGTTGACCCAAT | 198 | 95.0 | 1.0000 |
| COX1 | KT827242.1 | F: GAGACGACCAAATCTACAA  R: AAGTCAGTTACCGAATCCT | 101 | 102.0 | 0.9998 |
| GCG | NW_005393853.1 | F: GTGGCTGGATTGTTTGT  R: ACTTATCTTGGGCTGTATT | 136 | 97.0 | 0.9968 |
| MyoG | [XM_005895606.1](https://www.ncbi.nlm.nih.gov/entrez/viewer.fcgi?db=nucleotide&id=555967239) | F: TCTATGACGGGGAGAACTACCTG  R: CCTCTTACACACCTTACACGCC | 179 | 106.0 | 0.9983 |
| MyHC I | [XM_005909803.2](https://www.ncbi.nlm.nih.gov/entrez/viewer.fcgi?db=nucleotide&id=942097562) | F: AGGACCAAATGAATGAGC  R: GCTGGGTATAGGTGAGCTTG | 174 | 102.0 | 0.9978 |
| MyHC IIB | [XM_005907201.1](https://www.ncbi.nlm.nih.gov/entrez/viewer.fcgi?db=nucleotide&id=555990948) | F: TGACATTGACCACACCCAG  R: TCTCGCATCTCCTCTAGAATT | 87 | 98.0 | 0.9972 |
